# Driving ability after right-sided puncture of the common femoral artery during coronary angiography

**DOI:** 10.1007/s00392-018-1257-8

**Published:** 2018-04-19

**Authors:** Christoph Brenner, Raoul Fuehring, David Niederseer, Rudolf Kirchmair, Christian Haid, Michael Liebensteiner

**Affiliations:** 1 Department of Internal Medicine III, Cardiology and Angiology, Medical University of Innsbruck, Anichstr. 35, 6020 Innsbruck, Austria; 2 Department of Cardiology, University Heart Center Zurich, University of Zurich, Zurich, Switzerland; 3 Department for Orthopaedic Surgery, Medical University of Innsbruck, Innsbruck, Austria; 4 Reha Zentrum Muenster and Karl Landsteiner Institute for Interdisciplinary Rehabilitation, Tyrol, Austria

**Keywords:** Driving ability, Brake reaction time, Cardiac catheterization, Femoral puncture

## Abstract

**Objectives/background:**

To assess brake reaction time (BRT; key factor in driving ability) in patients receiving transfemoral coronary angiography (CAG). We assumed that patients would have a significantly impaired BRT after the procedure.

**Methods:**

A prospective, observational study design was applied. Consecutive patients undergoing right-sided transfemoral CAG as part of the clinical routine were included. An experimental driving simulator was used to determine BRT after receiving a visual stimulus. The subjects applied the brake with their right foot as quickly as possible when a red-light signal appeared. The time interval between stimulus and brake application was taken as BRT. In addition to the total BRT, also its components were determined: neurologic reaction time, foot transfer time and brake travel time. BRT was determined before and 1 day after CAG (pre-post comparison).

**Results:**

71 patients were included in the analysis (58 male, age 61 ± 9 years). Total BRT was 594 ± 188 and 591 ± 198 ms before and after the CAG procedure, respectively (*p* = 0.270). Similarly, also the BRT components ‘foot transfer time’ and ‘brake travel time’ did not show significant differences between the two test occasions. However, neurologic reaction time decreased from 269 ± 67 to 255 ± 64 ms (*p* = 0.036).

**Conclusions:**

We found no impairment of BRT on the first day after puncture of the right-sided femoral artery in patients undergoing CAG. Therefore, with regard to BRT, it is regarded safe to resume driving from day 1 after CAG. Other factors of driving safety beyond BRT must also be considered.

## Introduction

In the context of standard procedures of the lower limbs, physicians are often confronted with questions regarding driving ability after invasive procedures. Such questions are of obvious importance for patient safety as well as for the safety of other traffic participants. Driving ability is crucial for an individual’s participation in contemporary social life and an important task in activities of daily living. Therefore, an excessive driving prohibition following interventional or surgical treatment is not useful. When weighing the interests of safety vs. independence, it would be beneficial to have specific scientific knowledge about postoperative driving impairments following invasive medical procedures.

Among the various factors contributing to driving ability (visual acuity, amount of sleep, etc.), previous studies reported “brake reaction time” (BRT) to be a key parameter (synonyms: driving reaction time, brake response time) [1–3]. BRT was defined as the time interval between a (visual) stimulus (e.g. red traffic lights) and the application of sufficient pressure to the brake pedal.

Previous research provided good evidence on driving ability in the context of elective orthopaedic surgery such as total joint arthroplasty [2–8], knee arthroscopy [9, 10] or spinal surgery [11–14]. Previous studies also investigated the influence of inguinal hernia repair surgery on BRT [15–17]. For many other invasive standard procedures related to the lower limbs the literature contains little or no evidence on a patient’s postoperative BRT or general driving abilities. To the best of our knowledge, no previous studies have investigated the influence of standard vascular surgical procedures (e.g. femoropopliteal bypass surgery) on driving ability. Likewise, inguinal puncture of the right-sided femoral artery (e.g. for endovascular coronary procedures) has not been investigated although pain in the groin might impair driving ability.

Therefore, the purpose of this study was to assess brake reaction time as a key factor in driving ability in patients undergoing puncture and temporary catheter sheath implantation in the right-sided femoral artery for invasive coronary angiography (CAG). We assumed that BRT might be significantly increased after the procedure in our patients (pre-post comparison, Hypothesis 1).

## Methods

### Participants

We designed a prospective, observational study that was approved by the Ethics Committee of our medical university (No. AN2016-0050 360/4.2 367/5.4 (3922a)) and was performed in accordance with the ethical standards laid down in the 1964 Declaration of Helsinki and its later amendments. Consecutive patients undergoing CAG as part of the clinical routine of our university hospital were considered for participation and included after granting written informed consent. The only exclusion criteria were: (a) acute coronary syndrome, (b) no driver’s licence, (c) known neurologic diseases that would impair BRT.

### Procedure

Patients with stable angina pectoris scheduled for elective CAG via a transfemoral route were included. All patients were pretreated with 100 mg Aspirin at least the day before and on the day of the procedure. For CAG, we gained vascular access to the right common femoral artery after local anaesthesia with 10 cc 1% lidocaine. Using the Seldinger technique we inserted a vascular access sheath (Cordis Avanti® introducer, sized 6 or 7 french) in the femoral artery. Patients undergoing PCI of a coronary artery received a 600 mg loading dose of Clopidogrel and 60–70 IE/kg of unfractionated Heparin immediately prior to guide wire placement. After PCI, administration of Aspirin and Clopidogrel was continued once daily in all patients until the end of the study. After CAG completion, we removed the sheath and closed the vascular access site using the FemoSeal™ vascular closure device (St. Jude Medical®). All patients additionally received an inguinal compression bandage and rested in bed for the following six hours before mobilization. This procedure was identical in all patients regardless if they were treated with PCI or not. Patients with inguinal hematoma (> 5 cm diameter), clinically apparent pseudoaneurysm (vascular murmur) or immobilizing pain at the vascular access site were excluded from the follow-up measurement. For study exclusion due to access site complications, we only used clinical assessments. If clinically indicated, color-coded Doppler sonography was used to guide the further treatment strategies.

### Outcome parameters

**Fig. 1:**
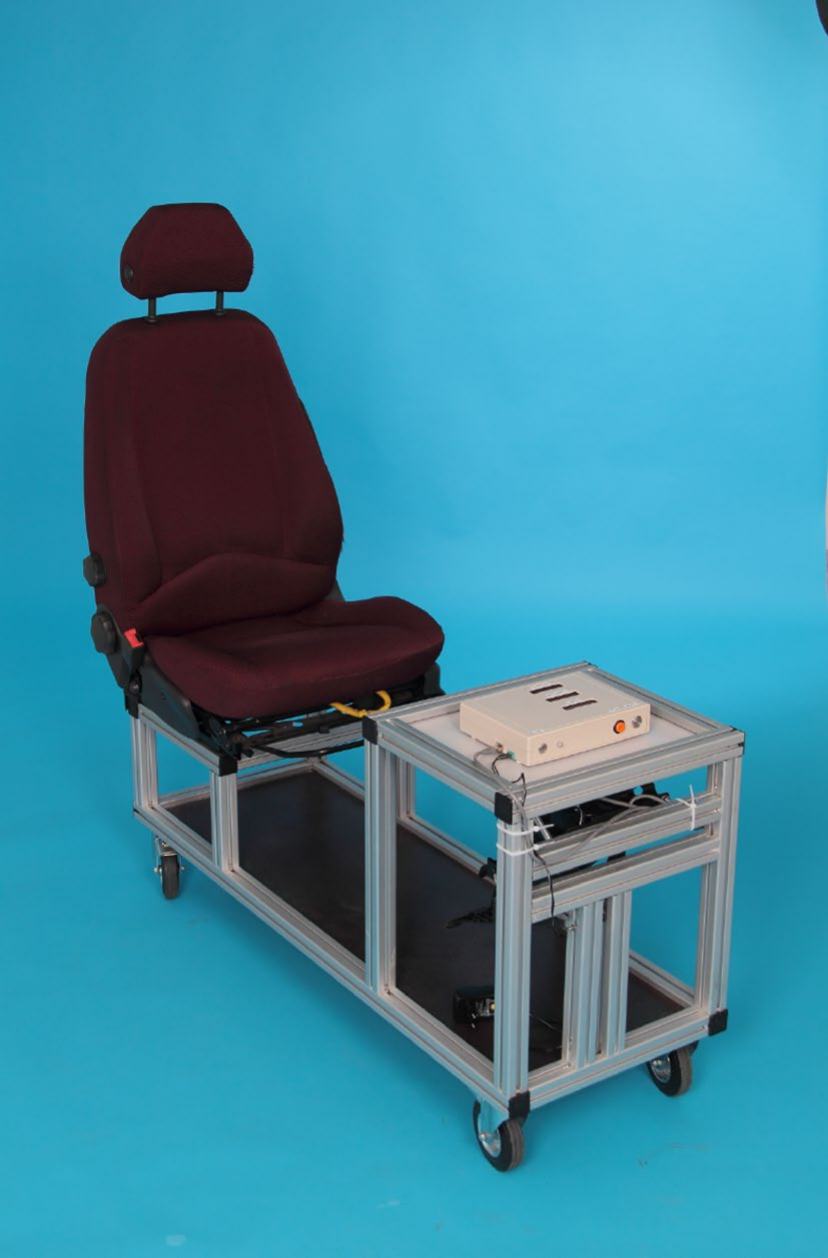
The driving simulator with an adjustable seat and the electronics. The trigger and a green lamp are orientated towards the observer

**Fig. 2:**
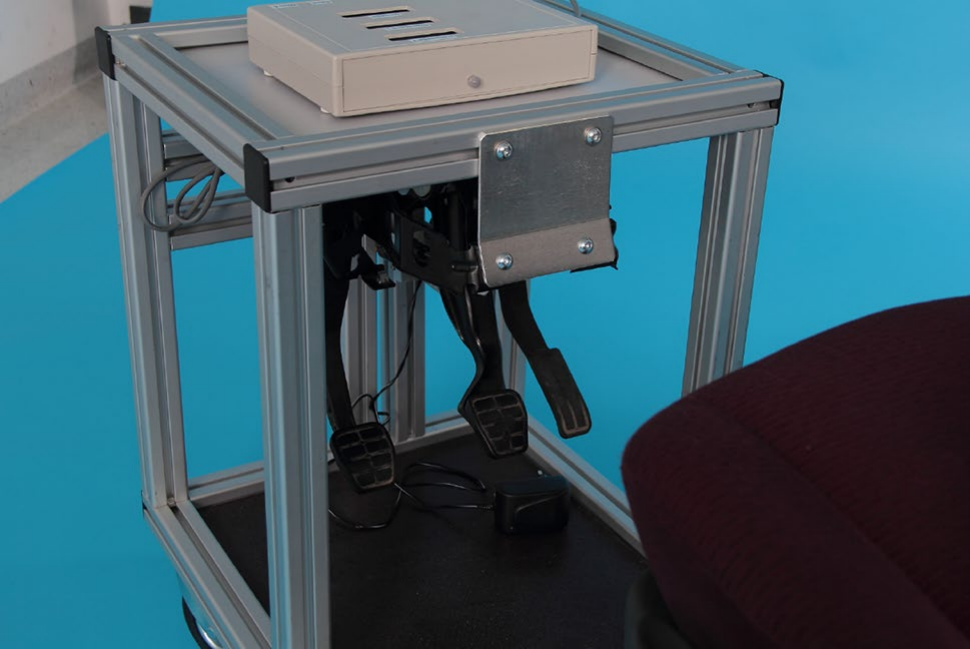
The box in front of the patient with a red lamp orientated towards the patient

Based on apparatuses described and validated in the published literature [1, 2], we devised an experimental apparatus for measuring BRT (Figs. 1, 2). An adjustable car seat was mounted on a frame with pedals hanging from rubber-damped pivots. The inclination of the seat, the head rest, the seat-to-pedal distance and the seat height were adjusted according to previous investigations [18], such that they resembled the patient’s usual driving position. An external suitcase containing the logic gate electronics, and a green and a red lamp was positioned on a table at a fixed distance.

The procedure was commenced by the patient fully depressing the accelerator, which was confirmed when a green lamp lit up. This prevented the patient from driving in a ‘ready-to-brake fashion’. After an interval of 5–10 s, the investigator pressed a switch concealed from the patient, which activated the red lamp (visual stimulus for the patient) and the electronic clock. Before starting the procedure, the subjects were instructed in a standardized manner to apply the brake with their right foot as quickly as possible when the red-light signal appeared. The time interval until the subject operated the brake pedal with 160 N was measured and taken as the BRT.

In addition to the total BRT, the integrated stopwatch also automatically determined individual components of BRT: neurologic reaction time (time from visual stimulus until the subject starts to pull back his foot), foot transfer time (time from start of foot retraction from the accelerator until initial contact with the brake pedal) and brake travel time (time from initial contact with the brake pedal until 160 N is reached).

Patients exclusively used their right leg to operate the pedals of the apparatus, while the left foot rested on the clutch pedal. After the subject had familiarized himself with the apparatus in three training runs, BRT was measured consecutively 10 times as described. An interval of 5 s was maintained between measurements. Total BRT and its components were calculated as the arithmetic average of the 10 measurements. All participants were given the same standardized instructions. BRT was determined before CAG to obtain a reference value. Another BRT measurement was performed in all patients on day 1 after CAG. On day 1 after CAG patients also rated their level of inguinal pain on a 10-point numeric rating scale (NRS).

### Statistical analysis

Data analysis was performed with SPSS (International Business Machines Corporation, Armonk, NY, USA). Data were not normally distributed as indicated by the Kolmogorov–Smirnov test. As descriptive values medians and interquartile ranges were determined. Wilcoxon tests were applied to test for significant differences in BRT between measurements 1 and 2. Alpha was defined as 0.05 (two-tailed).

## Results

Eighty-four patients were included and underwent BRT measurement at the first test. Of these patients, 13 did not participate in the second BRT measurement for the following reasons: organizational reasons: four, cancelled CAG: one, haematoma > 5 cm: three, immobilizing pain: three, refused: two. The remaining 71 patients were included in the analysis (58 male, age 61 ± 9 years). Patient characteristics are provided in Table 1.

**Table 1:** Patient characteristics

	Md	IQR
Age (years)	61	9
Weight (kg)	83.5	21.25
Height (cm)	174	11.25
BMI (kg/m^2^)	26.8	5.1

	Type	*n*
Type of procedure	Diagnostic	53
	Interventional	18

	Number	*n*
Stents implanted	0	53
	1	11
	2	4
	3	3

	NRS [0–10]	*n*
Inguinal pain on the day after CAG	0	55
	1	12
	2	3
	3	1

	Size	*n*
Sheath	6	67
	7	4

*CAG* coronary angiography, *Md* median, *IQR* interquartile range, *NRS* numeric rating scale

Total BRT was 594 ± 188 and 591 ± 198 ms before and after the CAG procedure, respectively (*p* = 0.270). Similarly, also the BRT components ‘foot transfer time’ and ‘brake travel time’ did not show significant differences between the two test occasions. However, neurologic reaction time decreased from 269 ± 67 to 255 ± 64 ms (*p* = 0.036) (Table 2).

**Table 2:** Descriptive and inferential statistics for total brake reaction time (BRT) and its components neurologic reaction time, foot transfer time and brake travel time

(ms)	Before CAG				After CAG				*p* value
	Md	IQR	Min	Max	Md	IQR	Min	Max	
Total BRT	594	188	373	1612	591	198	413	1891	0.270
Neurologic reaction time	269	67	174	577	255	64	174	576	0.036
Foot transfer time	224	80	156	545	228	85	147	707	0.703
Brake travel time	92	100	30	490	96	95	41	608	0.806

*CAG* coronary angiography, *BRT* brake reaction time, *Md* median, *IQR* interquartile range, *Min* minimum, *Max* maximum

## Discussion

The most important finding of the study was that BRT was not impaired on day 1 after puncture of the right-sided femoral artery. Therefore, it is deemed safe to resume driving from day 1 after CAG.

When trying to compare our findings with previous research, it appears that no such studies have been conducted to date. This is surprising because so many other standard medical procedures involving the lower limb have been investigated with regard to BRT. Those studies dealt with inguinal hernia repair [15–17], total hip arthroplasty [2, 5], total knee arthroplasty [3, 6, 8, 19], knee arthroscopy [10] and knee ligament reconstruction [9]. On the basis of their findings, those authors recommended postoperative driving abstinence as follows: 6 days after right-sided open inguinal hernia repair [16], 6–8 weeks after right-sided total hip arthroplasty [2, 5], 6–8 weeks after right-sided total knee arthroplasty [3, 8, 19], 1 week after right-sided knee arthroscopy [10] and 4–6 weeks after right-sided knee ligament reconstruction [9]. It is obvious that the above-mentioned procedures are much more invasive than puncture of the femoral artery with temporary catheter sheath implantation. Therefore, earnest comparison is not possible. Most of the above-mentioned studies used preoperative values as the safety reference, which may be due to the absence of an official reference value. Only some road authorities recommended a maximum BRT ranging between 700 and 1500 ms [20–22]. Other authors suggested that private drivers be allowed to resume driving 24 h after CAG, but recommended a 1-month driving abstinence for occupational drivers (lorry, bus, etc.) [23]. However, such recommendations are not based on scientific experiments.

As mentioned above, on the basis of our findings on BRT we do not recommend a ban on driving following right-sided puncture of the femoral artery with temporary catheter sheath implantation. Our findings might also be applicable to patients who undergo puncture of the right-sided femoral artery for endovascular therapy of peripheral arterial occlusive disease. Although we believe that also such patients should be allowed to resume driving, this must be viewed with more caution as we tested only patients who underwent CAG.

We did not analyze left-sided puncture of the femoral artery although invasive procedures of the left leg can potentially affect BRT, as was shown for total knee arthroplasty [7]. The exact mechanism underlying the effect of left-sided total knee arthroplasty on BRT is unclear. A plausible explanation was provided by Pierson et al. [3], who stated that the left leg performs a supportive function when transferring the right foot and is, therefore, involved in the process of braking. This presumed mechanism might be even more relevant in the dynamic circumstances of actual driving (such as negative acceleration during braking). However, as we did not find a significant impairment of BRT after right-sided puncture of the femoral artery, it is also deemed safe to resume driving after left-sided procedures, no matter whether automatic or conventional transmission is concerned.

We detected a slight decrease in neurologic reaction time in the post-CAG measurements compared to the baseline measurements. This difference was presumably based on a certain training effect as the assessments were conducted on two consecutive days. A difference of 14 ms was found, probably without actual clinical relevance. Additionally, experienced drivers would not experience the described training effect in their own car in real circumstances, i.e. no 14 ms delay due to the initial use of an artificial car simulator.

### Study limitations

The following limitations must be acknowledged. Thirteen patients could not be included in the second BRT measurement, thus giving a drop-out rate of 15%. Therefore, our results must be interpreted with caution. Moreover, the above-mentioned recommendations for patients with endovascular therapy of peripheral arterial occlusive disease must be viewed with even more caution as we tested individuals only following CAG. We studied driving safety only in terms of BRT. Although BRT was described as the most important factor [1], many other skills and factors are, of course, relevant for driving ability and were not investigated in our study [23]. As an example, the risk for stent thromboses after PCI may significantly impact on driving ability but is totally not reflected by the BRT assessed in this study. However, the study provides additional scientific knowledge in a field neglected by previous research.

The study findings are regarded as clinically relevant. No previous studies have investigated driving ability after the standard medical procedure of transfemoral coronary angiography. When weighing the interests of safety vs. independence, it is mandatory to have specific scientific knowledge about postoperative driving impairments following invasive medical procedures. Even if the transradial approach is increasingly used in CAG and BRT is very likely not affected by it, the transfemoral access in CAG is still widely used [24]. We, therefore, feel our results are still of relevance even though the transradial access in CAG is gaining ground.

## Conclusions

We found no BRT impairment on day 1 after puncture of the right-sided femoral artery in patients undergoing CAG. Therefore, with regard to BRT, it is deemed safe for patients to resume driving from day 1 following CAG. Other driving safety factors beyond BRT must also be considered.

## Abbreviations

BRT: Brake reaction time
CAG: Coronary angiography
NRS: Numeric rating scale
